# Comparison of monocular sensitivities measured with and without occlusion using the head-mounted perimeter imo

**DOI:** 10.1371/journal.pone.0210691

**Published:** 2019-01-17

**Authors:** Akemi Wakayama, Chota Matsumoto, Yoriko Ayato, Yoshikazu Shimomura

**Affiliations:** Department of Ophthalmology, Kindai University Faculty of Medicine, Osakasayama City, Osaka, Japan; Universidade do Minho, PORTUGAL

## Abstract

**Purpose:**

Using a head-mounted perimeter imo that can measure monocular sensitivity with both eyes open, we investigated the difference between monocular sensitivities measured with and without occlusion of the fellow eye and if the difference was influenced by eccentricity.

**Methods:**

Using the perimeter imo, monocular sensitivities with/without occlusion and binocular sensitivity were measured and compared. Three test conditions for monocular sensitivity without occlusion were: with/without a fusional fixation target, and a binocular random single eye test in which the target was randomly presented to either eye and the examinee was not aware of the tested eye. Within the central 25° visual field (VF), 29 points located at the fovea and on the 45°, 135°, 225°, and 315° meridians with 3° intervals were tested. Differences among the four monocular sensitivities with/without occlusion were further evaluated at the fovea, within and beyond the central 5° VF.

**Results:**

Sixteen visually normal volunteers (mean age, 28.6 ± 4.6 years) were included in this study. Except at the fovea, monocular sensitivities measured without occlusion were significantly higher than those with occlusion (*P* < 0.01). No significant difference was seen among the three monocular sensitivities without occlusion (*P* = 0.82).

**Conclusions:**

Except at the fovea, monocular sensitivities measured with and without occlusion significantly differed. This indicates that without occlusion, binocular interaction is activated and affects not only binocular sensitivity but also monocular sensitivity.

## Introduction

Clinical visual field (VF) testing is essential for diagnosing and following up neurological diseases. In VF testing, the non-tested eye is usually occluded to detect sensitivity loss because sensitivity compensation in the area with sensitivity loss could occur under binocular condition. During VF testing with occlusion, some patients however may experience darkening of the VF caused by binocular interactions such as blankout and binocular rivalry [1,2]. Blankout occurs when the two eyes experience different levels of illumination although it does not occur during binocular viewing [3]. Binocular rivalry occurs when different images are presented to the corresponding retinal areas of the two eyes. As a result, fusion becomes impossible and perception alternates between the different images [4–7]. The phenomena of blankout and binocular rivalry can cause uncertainty in VF sensitivity measurement.

Released in 2015, a head-mounted perimeter imo (CREWT Medical Systems, Inc.,Tokyo, Japan) developed by us helps solve the problems caused by the use of occlusion. In an imo test, the test target is randomly presented to either eye and the two eyes can be tested separately without occluding the non-tested eye [8]. Monocular sensitivity can be measured without occlusion and that is a technical innovation in VF testing. Since monocular sensitivity has always been clinically measured with occlusion, it is unknown if the sensitivities measured with and without occlusion differ. Moreover, it requires further investigation whether the condition of the two eyes sharing a uniform background and yet tested separately has any impact on the measured monocular sensitivity. With the perimeter imo, such investigation is possible.

Binocular sensitivity has been known to be higher than monocular sensitivity due to binocular summation (BS). Although previous studies on BS have compared binocular and monocular sensitivities measured with translucent occlusion [9–11], such comparison has not been made under a no-occlusion condition. Using imo, monocular and binocular sensitivities can be compared with the two eyes exposed to the same background. This could also help elucidate the mechanism of BS for higher binocular sensitivity.

This study aimed to examine: 1) Do monocular sensitivities measured with and without occlusion of the fellow eye differ? 2) If the monocular sensitivities measured with and without occlusion are different, is the sensitivity difference affected by eccentricity although sensitivity usually decreases with increasing eccentricity? We also compared binocular sensitivity with monocular sensitivity measured without occlusion to examine if our result was different from the previous results obtained with occlusion.

## Subjects and methods

### Subjects

Visually normal volunteers from our hospital were recruited. Inclusion criteria were as follows: age under 35 years old, best corrected visual acuity of 1.2 or better (-0.1 logMAR equivalent), refractive error < ±3.00 D (spherical) and < -0.75 D (cylindrical), normal stereopsis with 60 sec of arc or better, normal ocular alignment and ocular motility, and with experience in VF testing.

This prospective research was approved by the Ethics Committee of Kindai University (no. 26–239) and adhered to the tenets of the Declaration of Helsinki. Written informed consent was obtained from all the participants after explanation of the nature and possible consequences of the study.

### Sensitivity measurement

All the sensitivity measurement was performed using the imo. In an imo test, the target is presented on a full high-definition (HD) transmissive liquid crystal display with a high intensity light emitting diode (LED) backlight. The imo has a maximum target luminance of 3183 cd/m^2^ (10000 asb) and a background luminance of 10 cd/m^2^ (31.4 asb). The pupil is monitored independently for each eye and monocular sensitivity for the right or left eye can be measured without occluding the other eye. As the unique feature of the imo, the binocular random single eye test installed in the imo can randomly present a target to the right or left eye. Monocular sensitivities for the two eyes can be measured simultaneously in one single test without the examinee being aware of the tested eye. With the imo’s completely separate optical systems and stimulus presentations for the right and left eyes, a previous study has shown that the imo can obtain findings different from those by Goldmann perimetry and the Humphrey Field Analyzer in a patient with unilateral functional visual loss [12].

**Fig 1:**
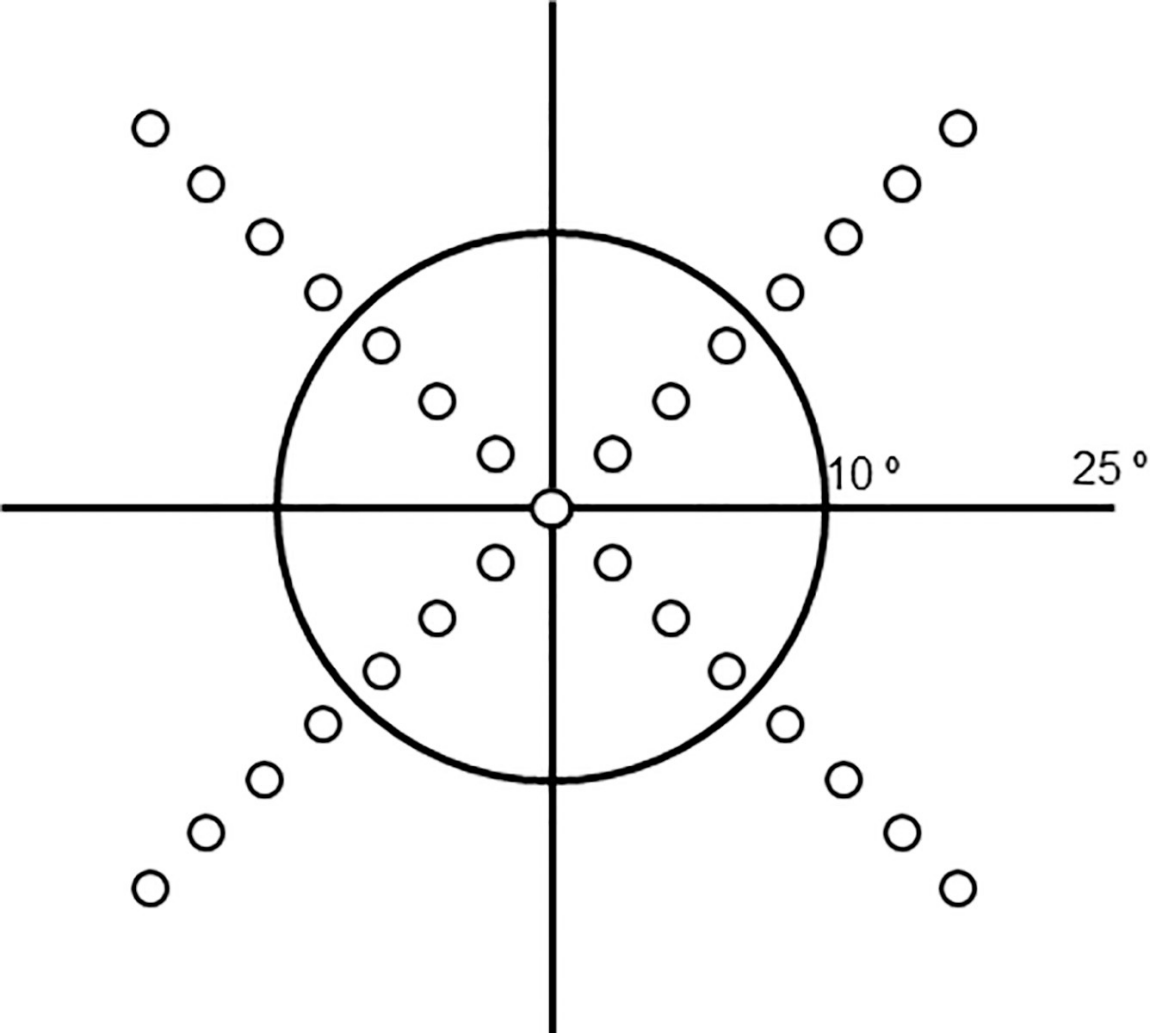
Schematic representation of the test locations. Located at the fovea and on the 45°, 135°, 225°, and 315° meridians with 3° intervals, 29 points were tested.

In this study, a total of 29 points located at the fovea and on the 45°, 135°, 225°, and 315° meridians with 3° intervals within the central 25° VF were tested using the Goldmann size Ⅲ (0.431° of visual angle) (Fig 1). The threshold algorithm used 4–2 dB bracketing strategy.

### Test conditions for monocular and binocular sensitivity measurement

**Fig 2:**
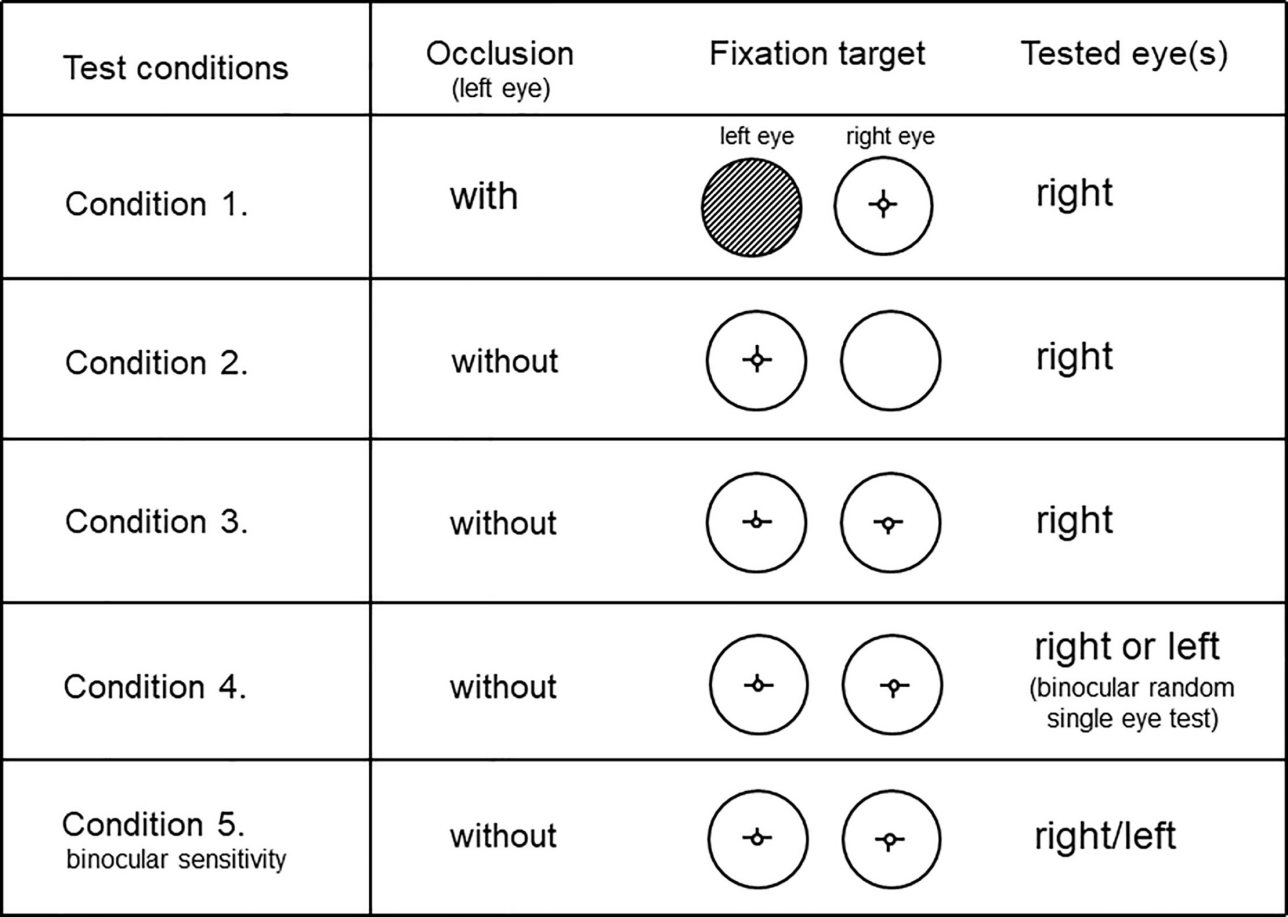
Test conditions for monocular and binocular sensitivity measurement.

In this study, monocular sensitivity used the measurement for the right eye and was obtained under four test conditions: one with occlusion of the non-tested left eye using a white opaque occluder (Condition 1) and three without occlusion (Condition 2, 3, 4). The differences among the three conditions without occlusion were the use of a fusional fixation target for the tested eye and the tested eye (only the right eye was tested or either eye was tested as in the binocular single eye test). Binocular sensitivity (Condition 5) was also measured (Fig 2).

Each condition was tested twice and the average of the two sensitivity measurements was used. Test condition and location were determined in a random order. Binocular sensitivity was compared to the best monocular sensitivity, which was the higher sensitivity of the two eyes in the binocular random single eye test. We also evaluated the sensitivity differences among the four monocular test conditions in respect to eccentricity (at the fovea, within 5°, and beyond 5°) to determine if the sensitivity differences were affected by eccentricity.

### Statistical analysis

Data were analyzed using BellCurve for Excel (Social Survey Research Information Co., Ltd). Sensitivity differences among the test conditions and at the three eccentricity ranges were analyzed by ANOVA and the Bonferroni/Dunn test. These statistical analyses were also used for the difference between binocular sensitivity and the best monocular sensitivity. Monocular sensitivity differences between the tests with the right eye only and the binocular random single eye test were analyzed by Wilcoxon signed-rank test. *P* < 0.05 was considered statistically significant.

## Results

Sixteen subjects [mean age, 28.6 ± 4.6 years; refractive error (sphere): - 1.31 ± 1.55 D (right), -1.13 ± 1.53 (left)] who met the inclusion criteria were included. The mean test durations for conditions 1, 2, 3, 4, and 5 were 3′33″ ± 25″, 3′39″ ± 26″, 3′01″ ± 24″, 6′16″ ± 58″, and 3′00″ ± 21″, respectively.

### Monocular sensitivities under the four test conditions and binocular sensitivity

**Fig 3:**
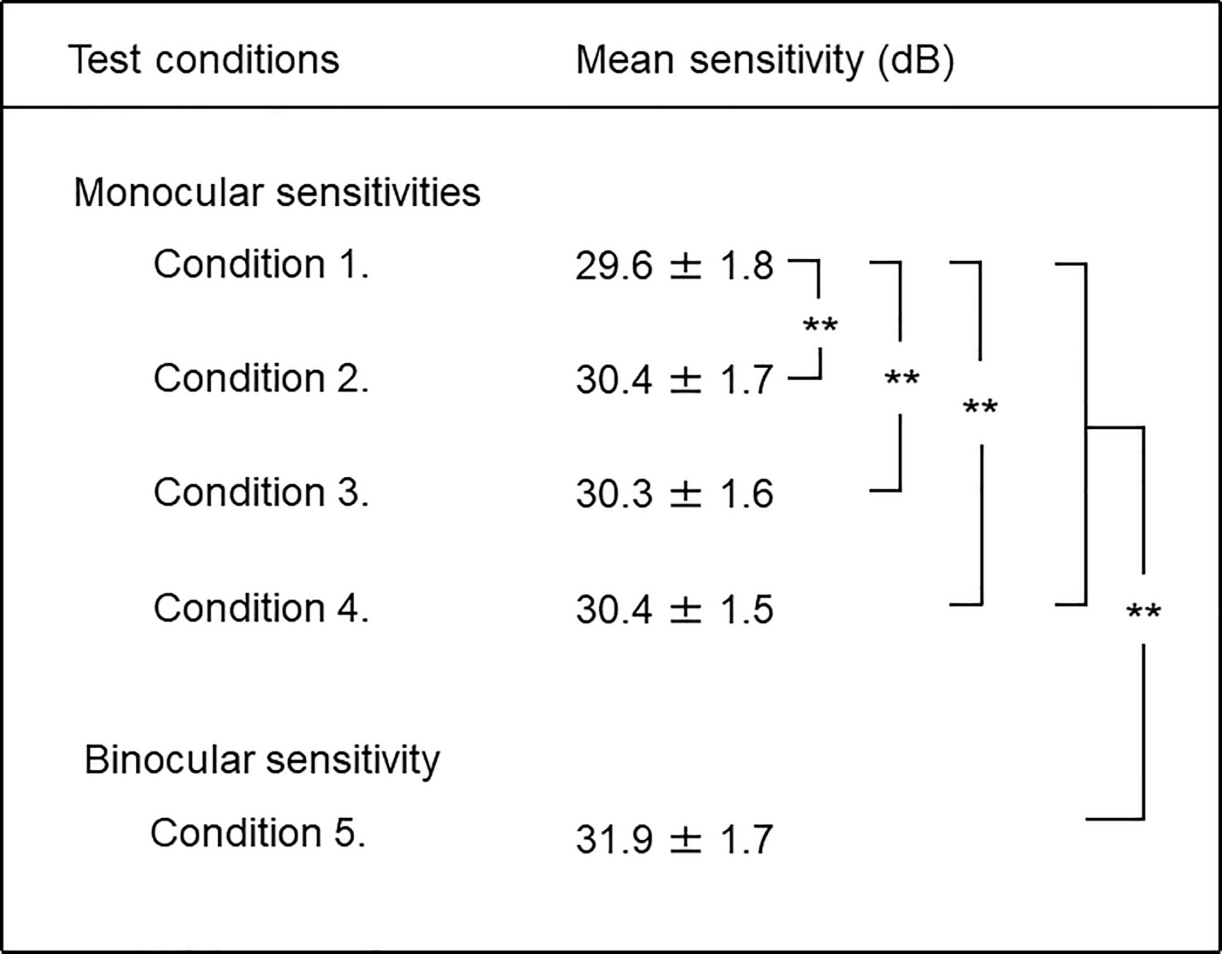
Monocular and binocular sensitivities. Data are mean ± SD for the mean sensitivity (**P < 0.01).

The three monocular sensitivities measured without occlusion were significantly higher than that with occlusion (*P* < 0.01, Fig 3 and Table 1). Among the three conditions without occlusion, monocular sensitivities with and without fusion did not significantly differ (*P* = 0.87). With fusion, the sensitivities for the right eye only and for either eye as in the random single eye test did not differ significantly (*P* = 0.82). Binocular sensitivity was significantly higher than the four monocular sensitivities (*P* < 0.01).

**Table 1:** Mean differences and confidence intervals for the sensitivity differences between the two indicated conditions.

Two conditions	Mean difference (dB)	95% confidence interval
Condition 2 and 1	0.82	0.65–0.99
Condition 3 and 1	0.75	0.58–0.92
Condition 4 and 1	0.82	0.63–1.02
Condition 3 and 2	-0.07	-0.18–0.03
Condition 4 and 2	0.00	-0.18–0.19
Condition 4 and 3	0.08	-0.02–0.18
Condition 5 and 1	2.31	2.11–2.52
Condition 5 and 2	1.49	1.33–1.66
Condition 5 and 3	1.57	1.42–1.73
Condition 5 and 4	1.49	1.29–1.69

### Sensitivity differences among the four monocular conditions at the three eccentricity ranges

**Fig 4:**
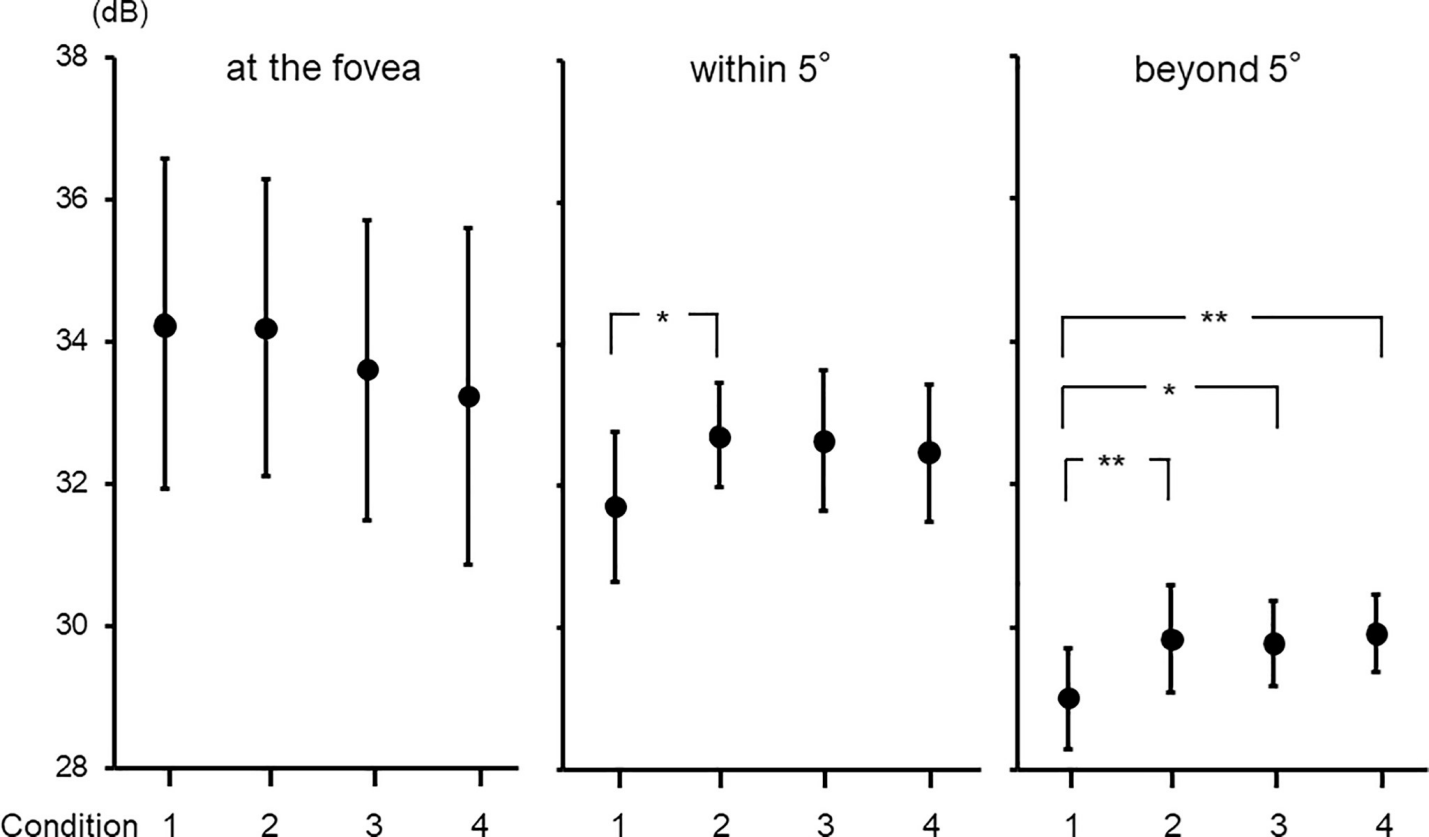
Sensitivity differences among the four monocular conditions at the three eccentricity ranges. Beyond 5°, monocular sensitivities without occlusion were significantly higher than the sensitivity with occlusion (**P < 0.01 and *P < 0.05). Data are mean ± SD for the mean sensitivity.

Beyond 5°, the three monocular sensitivities without occlusion were significantly higher than that with occlusion (*P* < 0.01 for the differences between Condition 1 and 2, and 1 and 4; *P* < 0.05 for the difference between Condition 1 and 3; Fig 4 and Table 2). At the fovea, the monocular sensitivities with and without occlusion did not significantly differ (*P* = 0.53). While the sensitivity with occlusion was the lowest outside the fovea among the four monocular sensitivities, it was the highest at the fovea.

**Table 2:** Mean differences and confidence intervals for the sensitivity differences between the two indicated conditions for the three eccentricity ranges.

	Two conditions	Mean difference (dB)	95% confidence interval
Fovea	Condition 2 and 1	-0.06	-1.79–1.67
	Condition 3 and 1	-0.66	-1.34–0.03
	Condition 4 and 1	-1.03	-1.90–0.16
	Condition 3 and 2	-0.60	-2.23–1.04
	Condition 4 and 2	-0.97	-2.60–0.66
	Condition 4 and 3	-0.38	-1.31–0.56
Within 5°	Condition 2 and 1	0.58	0.21–0.95
	Condition 3 and 1	0.86	0.35–1.37
	Condition 4 and 1	0.48	0.21–0.74
	Condition 3 and 2	0.28	-0.12–0.68
	Condition 4 and 2	-0.10	-0.30–0.09
	Condition 4 and 3	-0.38	-0.66 –-0.10
Beyond 5°	Condition 2 and 1	0.83	0.37–1.29
	Condition 3 and 1	0.78	0.44–1.11
	Condition 4 and 1	0.91	0.58–1.25
	Condition 3 and 2	-0.05	-3.01–0.20
	Condition 4 and 2	0.09	-0.21–0.38
	Condition 4 and 3	0.14	-0.07–0.35

### Comparison between binocular sensitivity and the best monocular sensitivity

**Fig 5:**
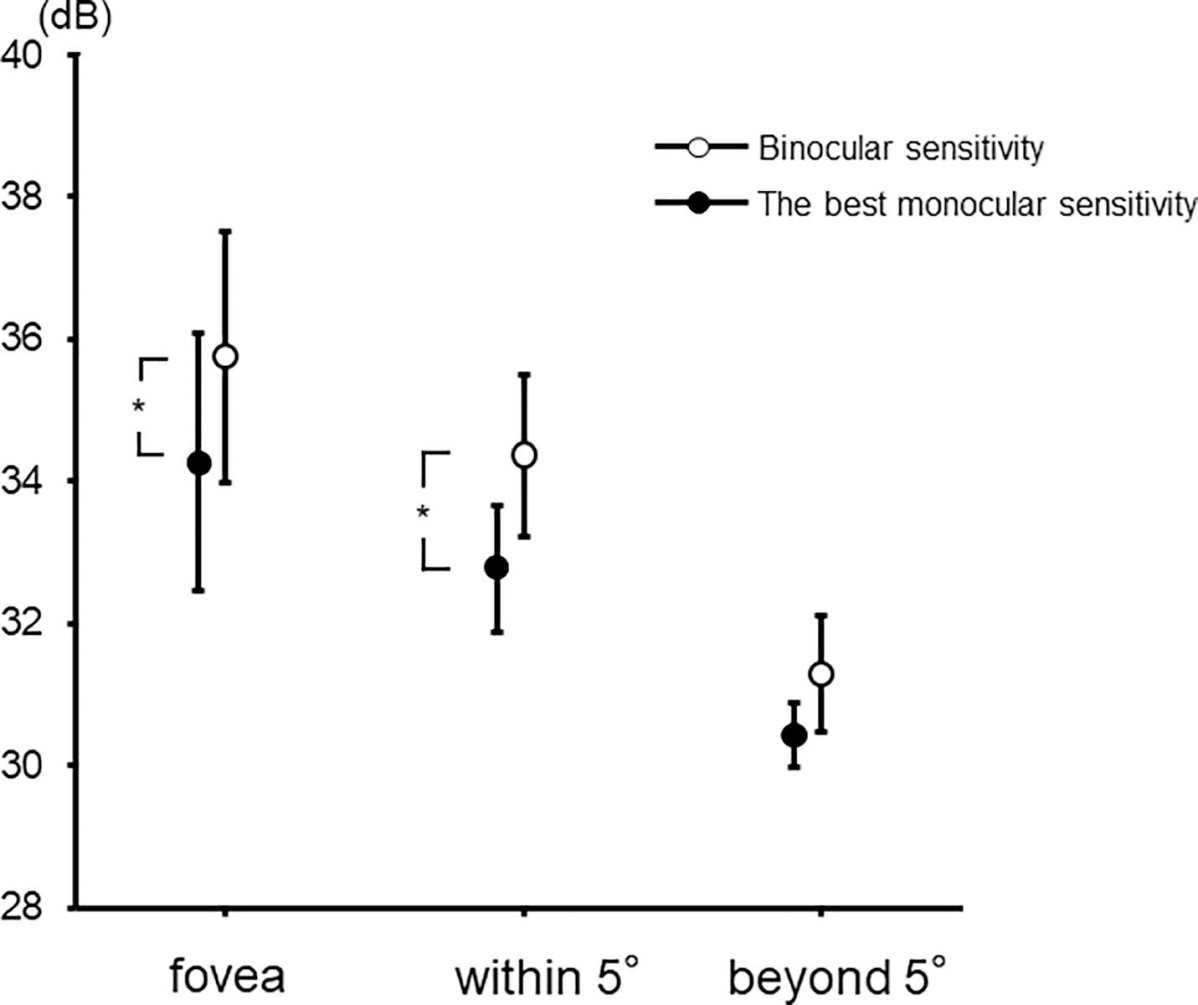
Comparison between binocular sensitivity and the best monocular sensitivity. At the fovea and within 5°, binocular sensitivity was significantly higher than the best monocular sensitivity (*P < 0.05). Data are mean ± SD for the mean sensitivity.

Binocular sensitivity was significantly higher than the best monocular sensitivity at the fovea and within 5° (*P* < 0.05), but not beyond 5° (Fig 5).

## Discussion

This study has demonstrated that overall, monocular sensitivity measured without occluding the non-tested eye was significantly higher than the sensitivity measured with occlusion. However, no difference was seen at the fovea. This indicates that without occlusion, the function of binocular interaction can be activated by presenting a uniform background to both eyes and affects the monocular sensitivity.

When monocular sensitivity is measured with the non-tested eye occluded, darkening of the eye (or blankout) may occur [1–3] and affects the sensitivity measurement. Blankout occurs when both eyes simultaneously experience different levels of illumination [3]. Visual disturbances such as blankout do not appear to be affected by ocular dominance [2]. Reportedly, monocular sensitivity measured with a translucent occluder is higher than the sensitivity measured with an opaque occluder and global indexes are also improved [1,13]. These studies suggest that the test condition of occluding the non-tested eye may lower the monocular sensitivity for the tested eye. Other studies also showed that the threshold of the second eye tested is higher than that of the first eye using frequency doubling perimetry, and this effect can be minimized using a translucent occluder [14,15]. These results indicated that light adaptation in both eye is important for perimetric threshold measurement. By eliminating the test condition of occlusion in this study, we considered that the effects of blankout and the light adaptation difference between both eyes had been avoided and resulted in an overall higher monocular sensitivity without occlusion.

Another explanation for the overall higher monocular sensitivity without occlusion may be the activated function of binocular summation. We have previously reported that binocular summation increases when monocular perception becomes more difficult with smaller stimulus size [10,11], lower contrast [16], more difficult recognition tasks [17], or more complex backgrounds [18]. Moreover, the amount of binocular summation increases in the peripheral area [17,18]. The current result showed that the difference between the monocular sensitivities measured with and without occlusion significantly increased beyond 5° of the VF. We therefore considered that without occlusion, the tested and non-tested eyes were exposed to a uniform background and binocular summation functioned and affected the monocular sensitivity difference beyond 5° although only the tested eye was stimulated by the test target.

On the contrary, the monocular sensitivity difference was not significant at the fovea. Like stereopsis, binocularity highly functions at the fovea and binocular rivalry can occur when different stimuli are presented to the foveae of the two eyes. Although both eyes were equally stimulated by the same background in the imo test, the test target was only presented to the tested eye and this might have caused binocular rivalry and lowered the monocular sensitivity without occlusion at the fovea. Reportedly, when the stimulus contrast for the two eyes is reduced to a low contrast level near the threshold, binocular rivalry does not occur but binocular fusion does [19]. In this study, binocular rivalry did not occur beyond 5°. We suspected that the likelihood for binocular rivalry to occur may vary depending on the region of the retina. That is, binocular rivalry may be more likely to occur at the fovea than in the periphery. Our current results suggested that over the VF, binocular interaction might have exercised influence differently on the monocular sensitivity measured without occlusion, involving binocular rivalry at the fovea and binocular summation in the periphery.

The present study however has several limitations. In order not to further complicate the test conditions, only the measurement for the right eye was used for monocular sensitivity. As shown in our previous studies, no significant sensitivity difference is observed between the two eyes in visually normal adults [10,17,18]. We therefore considered that using the measurement for the right eye as monocular sensitivity would be adequate. However, the possible effect of eye dominance on the measured monocular sensitivity with both eyes open should be further investigated. In addition, this study only included healthy eyes. Since some of the sensitivity differences were smaller than 1 dB, such small differences may not have a big clinical impact on normal eyes and the potential use of the imo in a clinical setting may require further validation. In the future, we are planning to use the imo on eyes with various VF defects to investigate if the difference between the sensitivities with and without occlusion varies with the severity of the VF defects, location of the defects, and unilaterality/bilaterality of the defects.

## Conclusions

Except at the fovea, monocular sensitivities measured with and without occlusion significantly differed. This indicates that without occlusion, binocular interaction is activated and affects not only binocular sensitivity but also monocular sensitivity.

## Supporting information

S1 File Supplementary data of this study. (XLSX)
